# "They Are After Quantity, Not Quality": Health Providers’ Perceptions of Fee Exemption Policies in Morocco

**DOI:** 10.15171/ijhpm.2018.76

**Published:** 2018-08-26

**Authors:** Karen Van der Veken, Fahdi Dkhimi, Bruno Marchal, Peter Decat

**Affiliations:** ^1^Department of Public Health, Institute of Tropical Medicine, Antwerp, Belgium.; ^2^Department of Public Health and Primary Care, Ghent University, Ghent, Belgium.

**Keywords:** Health Workers’ Motivation, Morocco, Exemption Mechanisms, Healthcare Reform, Policy Implementation

## Abstract

**Background:** A free obstetric care policy (FOCP) has been implemented in Morocco in 2008 in order to further decrease maternal mortality.

**Methods:** Through in-depth interviews we explored the perceptions of health professionals in public Moroccan hospitals with regard to fee exemption policies. We tried to understand what drives health professionals to ignore, modify or apply a health policy as formulated.

**Results:** Respondents express significant influences of such policies on their work environment (higher workload and scarcity of resources) and on the patient/provider relationship, both of which may cause a negative effect on health workers’ motivation. A mix of motivational determinants incites health workers in their turn to influence policy implementation.

**Conclusion:** Understanding the motivational determinants of health workers may optimize policy implementation at the point of service delivery

## Background

In 2002, Morocco passed a Basic Health Coverage bill aimed to improve financial protection for the whole population in case of access to health services. This law established a contributory Health Insurance regime for the population able to contribute financially into such a system, relying on insurance principles. It targets formally employed individuals, students, (ex)militaries and those entitled to a pension. This regime was made compulsory for formal employees in 2005 (AMO), through the establishment of two distinct schemes: one for the employees of the public and one for those of the private sectors. In 2016, the student scheme was introduced, managed under the civil servants’ national insurance scheme. The law made also special provisions for the economically deprived population through the establishment of a Medical Assistance Regime, the *Régime d’Assistance Médicale* (RAMED). This regime emerged from longstanding discussions as the optimal option to guarantee financial protection to vulnerable populations. After 3 years of piloting, the RAMED scheme was introduced nationwide in 2012.^1^

Despite these provisions, the Moroccan government felt the urge to take additional actions to improve health coverage through targeted fee exemptions in the public health sector. As maternal mortality reduction has been a top priority for Moroccan policymakers for several years,^2^ the government introduced a free obstetric care policy (FOCP) in 2008 in order to accelerate the country’s progress towards the Millennium Development Goals (MDGs). The FOCP aims to make obstetric and neonatal care in public hospitals accessible for all pregnant Moroccan women, regardless their socioeconomic status.^3^ The FOCP benefit package has been adjusted over time and currently exempts pregnant women from most medical costs incurred at the point of service for delivery, potential obstetric complications and caesarean section services, as well as for some of the indirect costs related to transport services for referral. The Ministry of Health compensates public hospitals through in kind and credit lines for drugs and supplies through the provision of delivery kits, vital drugs and blood products, a standard blood test and credit for fuel the transport between health facilities.^3^ These allocations are subject to the number of services provided to the population. As such, the FOCP – though not the only, nor the first exemption policy in Morocco – is the first FHC policy to compensate the public service providers for the services rendered to the population.

Intuitively, making services free of charge improves health coverage. Studies, however, show that FHC policies do not necessarily lead to the intended effects, due to multi-faceted implementation gaps. Evidence suggests that while financial barriers are significant, on their own their reduction does not change behaviour, unless it is entrenched in a multipronged strategy that aims for positive shifts in other aspects, such as perception of quality and responsiveness.^4^ Numerous publications focus on contextual determinants of policy implementation gaps.^5-8^ Health (and other) policies are often decided upon at central level and executed in a top-down manner. This implies that all the way down to the implementation, clear objectives and a precise description of the policy’s modalities are needed, in written guidelines and standardized operational procedures. If absent, managers and health professionals lack uniform and clear info, and the policy risks to not be implemented as planned. To improve the implementation of health policies, all actors of the health system need to be concerted and collaborate from the conception of the policy onward and all along the implementation process.^8^

Yet, more than only process-related factors influence the effectiveness of policy implementation. The FEMHealth research project (2011-2014) assessed the effectiveness of free obstetric care policies in Benin, Burkina Faso, Mali, and Morocco. It explored the intended and intended consequences of these policies and how they impact, positively and/or negatively, local health systems.^4,9^ A striking phenomenon across these countries was the various distance between design and implementation. These differences existed within a given country, showing a strong variability in the implementation of a policy. Also across countries, such gap was systematically observed, regardless of the difference in the policy design, suggesting that the broader political context and the processes of policy formulation and implementation alone do not determine the effectiveness of policy implementation. In some sites of Benin, for example, women still paid high fees for accessing caesarean section, while compensation for caesarean delivery to the providers was proved to be sufficient to cover the actual costs.^10^ Similar observations were made in Burkina Faso, despite the fact that the FHC design differs in the two countries.^11^

FEMHealth findings and several peer reviewed studies provide evidence for Lipsky’s *street level bureaucracy theory*: although they come last in a long hierarchical line, public servants are not powerless.^12^ They have a certain discretionary power allowing them to modify the implementation of a new policy in their best interests, with fluctuating consequences for the target population.

Unlike in Benin and Burkina Faso, the Moroccan FEMHealth study sites did not provide evidence of local differences in policy implementation. This raised a number of questions, among others with regard to the impact of the political and cultural context of a country on the extent and the use of discretionary power.

In this paper, we present the results of a study that aimed exploring the distance between what is formulated at central level and what eventually is implemented by the health professional. It addressed the question: what drives a professional to ignore, modify or apply a policy as formulated? We sought answers through a qualitative study to explore the perspectives of health professionals in Moroccan hospitals with regard to FHC policies. The study results may lead to optimization of health policy implementation, in order to maximize chances on achieving the planned objectives, which is what effective implementation actually entails.^13^

## Methods

This is a qualitative study in which data were collected through in-depth interviews and analysed using a thematic analysis approach. The authors obtained ethical approval from their institutes.

### Data Collection

Data were collected in seven hospitals distributed over six Moroccan health provinces or *Délégations Sanitaires Provinciales (DSP*), namely Tetouan, Settat, Al Haouz, Sidi Kacem, Kenitra and Marrakech. We selected sites that differed in service utilization, population concentration and poverty index.^14^

We contacted the regional health officer who sent an official letter to the selected hospitals, informing the director of the study objectives. They informed midwives, doctors and supervisors in the maternity of the potential request to participate in the study, before or after their working hours.

The interviews took place on-site – except two that were done outside the work place – in the third week of July 2016. All interviews were conducted in French, with a French-Berber interpreter assisting in one interview and a French-Arabic interpreter in one other. Prior to the interview, respondents were asked for a written consent and permission to record the interview. Notes were taken simultaneously. A semi-structured interview guide was used and following topics – plus any other emerging from the interview – were discussed: policy information and implementation modalities, ethical and professional appreciation of FHC, and perceived changes in the work environment, in the patient-provider relationship and in the work motivation. The average duration of an interview was 45 minutes.

### Data Analysis 

Data were analysed manually and thematically. A thematic diagram (Figure) was constructed in a theory-driven manner.^15^ The themes’ choice was inspired by peer-reviewed literature^5,7,16-19^ and FEMHealth results.^9,14,20^

**Figure:**
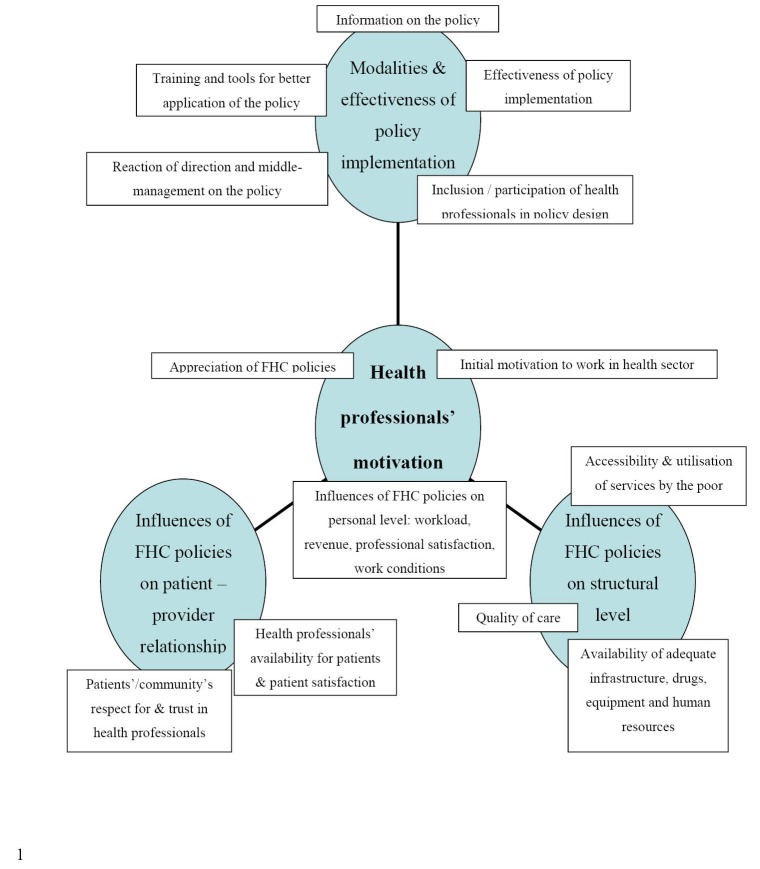
Thematic Diagram.

### Validity

In order to increase the credibility of this study, several procedures were used following standards of qualitative research.^21,22^

Amongst other, data were triangulated making use of different sources, namely interviews of staff with different functions, hospital statistics and the reports of earlier studies in the same hospital settings (FEMHealth project). When respondents had an outlier explanation for a phenomenon we encouraged them to go beyond the questions of the interview guide. Interpretation bias was avoided by discussing the coding among three researchers and by using two different theoretical frameworks: Lipsky’s Street Level Bureaucracy theory and Franco’s theory of motivational influences on health professionals.

## Results

Before presenting the results, we clarify that the RAMED policy was scaled up after the FOCP, but respondents tend to mention RAMED in the same breath as the FOCP when discussing the effects of FHC policies. We chose not to differentiate when not done so by the respondents. We took into account their perception of all FHC policies. In principle though, the RAMED card does not serve within the walls of the maternity.

### Characteristics of Respondents and Their Initial Motivation to Work in Health Sector

We interviewed 19 health professionals of whom 14 are women. Three work in a university hospital, five in provincial and ten in regional hospitals. Fifteen provide healthcare on a daily basis: seven nurse-midwives, one gynaecologist, and seven nurse supervisors. Further, four high-level managers were interviewed, upon their explicit request to be included as interviewee. The average professional experience of the respondents is 12 years (based on information from 13 respondents) and respondents are, on average, nine years functional in the current workplace (based on information from 16 respondents). For most of the study respondents the gratifying aspect of caring for people has been a major reason to choose for a job in the health sector. Another motivating factor is the professional security offered by a job in the public sector. Most respondents for whom the job was not a vocation, state to have learned to appreciate the task little by little. “*I did not choose by love. Now I love this profession, but I really don’t like the work conditions, they are not satisfying*” (R11).

### Modalities and Effectiveness of Policy Implementation

Health providers acknowledge that the FOCP was clearly formulated on paper, detailing the exempted package of care. Yet, they report a lack of training and guidelines for those that are involved in the practical implementation of the policy. Several professionals argue that their knowledge of the policy was mainly based on what they heard in the media. Regarding public sensitization campaigns in general, some respondents state that they were not sufficiently adapted to the vulnerable, being mostly illiterate and unaware of Moroccan politics.

“*They might have heard some things, but have not been explained their rights. We still receive people from rural areas who are entitled to a RAMED card, but who don’t have any. It is poor sensitization*” (R18).

While hospital directors wonder who will pay for FHC given the fact that maternities are traditionally revenue-generating departments within hospitals, respondents are unanimous: hospital directors have put in place the FOCP the day after the ministerial letter’s arrival. Yet respondents enumerate several exceptions on free obstetric care today. First, at tertiary level, women pay for all medical acts, including caesarean sections, if they do not present a referral letter or RAMED card. Second, gynaecology acts, ultrasound and curettage inclusive, are not exempted. Third, stock ruptures oblige parturients to buy drugs and supplies themselves in many cases. Fourth, transport between first and higher level of care is often paid for by the patient because of insufficient gasoline for the ambulance.

Also for holders of the RAMED card, exceptions on FHC exist. Moreover, they are explicitly formulated in the RAMED policy, stating that the RAMED covers care for the poor and vulnerable “within the resources available.” Consequently, many beneficiaries are asked to buy supplies and drugs, or to go elsewhere for further examination.

These exceptions on FHC demonstrate that policy implementation has been partly impeded by the policymakers to start with:

*“The support to the policy was not as such that service is available everywhere. In the beginning, there was financial space, so many people benefited from service. Now both policies start their last breath and are declining”* (R14).

Where FHC policies are not implemented as supposed to, respondents seek the cause in a deficient mobilization of resources, which leads to care seekers and caretakers being urged to look for solutions that become in their turn an embedded problem:

“* Corruption has a simple rule. When the means are limited and the demand is unlimited, you need money to have a bed, a service. That is how it works everywhere in the world. So corruption became structural” (R14).*

Several respondents argue that a more participatory approach would have benefited the implementation of the policies. Health professionals were consulted but did not actively take part in the design of FHC policies. When asked what could be success factors in health system reform, respondents reply that participation and engagement of all stakeholders (managers, providers, users) is key:

“*During our studies we have learned that, to succeed in policy change, one needs to involve the people” (R2).*

Prior to exploring providers’ appreciation of FHC policies, we acknowledge the context in which Moroccan health professionals implement health policies designed at central level. The FOCP started off as priority number one for the government under the guidance of the female minister for whom the women’s cause was almost a personal matter.

*“There was fuss, field missions and inspections. Some hospital directors were fired as a consequence of maternal deaths, unavailability of blood, communication problems, unworthy referral conditions for patients… Automatically, this policy became a priority for everyone”* (R6).

Appreciation of FHC Policies: “Politics Rather Than Good Management of Health”

Acknowledging that neonatal and maternal mortality has a lot of determinants, respondents doubt the effectiveness of the FOCP in reducing maternal and neonatal mortality:

*“Whether women come or not, it depends on how far they live, that is all”* (R7).

According to respondents, quality of care remains poor, or even has deteriorated due to an influx of patients and a lack of input in all building blocks of the health system.

*“These policies are not national priorities: they [policy makers] are after quantity, not quality”* (R4).

Respondents also question the sustainability of these policies. They are unanimous: FHC policies are expensive and, considering the country’s budgetary deficiency and fragile economic context, not made to last.

Influences of FHC Policies on Personal (Health Professionals) Level

From an ethical point of view, health professionals feel somehow satisfied being able to provide FHC to care seekers. They speak of a humanitarian mission, which they consider part of public service. It feeds their intrinsic motivation. On the other hand, work satisfaction is said to have “taken a free fall” because of decreasing quality of care, worsening working conditions, the feeling of being pursued rather than protected in the exercise of medical care and a lack of recognition for health professionals in general by both the authorities and the community.

A first negative association between FHC policies and health workers’ motivation is caused by deteriorating working conditions. Respondents unanimously consider workload to be substantially higher after the implementation of FOCP. As a consequence of the changing work environment, a negative effect is observed on the quality of care:

*“These policies can increase the number of patients seen but there is collateral damage with regards to quality, users’ satisfaction and even our own cost-efficiency”* (R5).

Secondly, and adding to the impression to work in unacceptable conditions, respondents mention a lack of recognition by the community and the authorities. Media sensationalize maternal death stories; health professionals and hospital managers are made responsible for a maternal death when it occurs on their watch, and risk to be fired and/or sentenced:

*“Look at the mediatization: they set the population against us. That is what the minister does”* (R4).

Increased stress on the work floor, increasing expectations of the community and the healthcare users, reinforced supervision by authorities and mediatization of medical errors create an environment of fear and mistrust. Health professionals feel pursued rather than protected in the exercise of medical care. The state is not seen as a partner or protecting agency, but as the one endangering the public agent.

*“We are not protected. Nothing motivates me. Why will I pay to clean up the mess when the state does not cover me? It is better for me to refer the women”* (R4).

Finally, despite deteriorating work conditions, FHC policies did not positively influence the revenue of health professionals. Public agents’ revenue only increases through seniority or by an evolution in grade after having succeeded in an exam.

Still, respondents describing their professional motivation as strongly intrinsic (being motivated by the idea of caring for people and making a true difference in the lives of some) argue that FHC policies have affected their professional satisfaction.

*“When practicing a profession with love, we give a lot; we forget all of the constraints of the policy”* (R9).

Influence of FHC Policies on Structural (Hospital and Services) Level

Respondents observe an influence of FHC policies at organizational level: service utilization has increased, which in turn led to an inadequacy of (human and material) resources, which in turn contributed to decreased quality of care and eventually to increased health inequity.

In Morocco, the institutional delivery rate increased significantly since the initiation of the FOCP, also among poor or vulnerable women, which was one of the major aims of the policy. Yet, respondents wonder whether increased access to healthcare of poor (or worsening) quality truly translates into increased accessibility:

*“The problem is that politicians are not interested in the quality of care, but only concerned by how many deliveries we assist. Accessibility has not really increased by this policy”* (R3).

Respondents state that health providers’ mentality has changed due to this focus on quantity:

*“It causes falsification of files. A woman dies and because of fear for complaints, we meet and create another file, a scenario which we discuss afterwards in the maternal death audit. If we exceed a certain number of deaths, we lose fees and credit. We cheat on the maternal death audits. It’s done everywhere, I think”* (R7).

No additional staff has been appointed in the aftermath of (or prior to) FOCP implementation – or at least not in correlation with the increased demand. Combined with cross-posts, sick leaves and retired professionals who were not replaced, this caused a relative scarcity of health providers. Under the FOCP, drug and equipment kits are provided, and in some places infrastructure was improved. However, the patient influx after the implementation caused stocks to be consumed rapidly, affecting mainly postpartum care:

*“Keeping women and newborns for 48 hours implies the need for 90 beds. We only have 60. This policy affects correct case management negatively. No means, no resources, no time, no beds, no space in the operation theatre… This is more or less due to the FHC”* (R15).

Respondents are explicit about the effect of FHC policies on the quality of care:

“*It is catastrophic. It’s impossible to talk about ‘safe motherhood’. It is unsafe motherhood. I have staff on duty since yesterday morning 8:00 am. It is logical that errors occur” (R7).*

The conditions described above cause a certain degree of stress and fear, eg, to be pursued in justice.

Furthermore, some respondents indicate a segmentation of the health system since FHC policies have been introduced, with public hospitals becoming hospitals for the poor and wealthy people going to private clinics. This would imply more health inequity, instead of less as aimed for by FHC policies. Others argue that even for the poor, the public hospital is the last resort. They prefer to put themselves into debt in order to find quality care in the private sector. Healthcare users, wealthy and poor, are said to practice cherry picking: they take what is free in public facilities and go in the private sector for all what is to be paid for.

Influences of FHC Policies and Patient (Community)/ Provider Relationship: “We Are Social Workers Now, Rather Than Healthcare Providers”

Respondents argue that FHC policies have generated high expectations in the community, but also that it had a negative influence on the patient/provider relationship.

First, according to respondents, patients consider conditions of admission and hospitalization as not satisfying. For many, such negative experience contributes to a negative impression of the overall quality of care. Moreover, respondents believe that they have less time for patients since the implementation of FHC. Some strongly intrinsically motivated respondents try to mitigate this limited availability through better communication with the patients.

Second, respondents argue that healthcare users do not appreciate FHC because people feel useless or like a burden when receiving everything for free. Moreover, they say that FHC is considered to be care of low quality, a perception that is reinforced by the impressions patients have upon admission in the hospital. Some respondents state to have been asked by patients to “be allowed to pay” in order to have better quality care. In the same logic, a health manager explained that the hospital bills are presented to RAMED patients, in order for them to see what has been exempted:

*“Those praising the RAMED are those who received care for serious or costly diseases or surgical interventions; people talking badly about it are the others”* (R6).

Finally, several health professionals come to the conclusion that FHC policies strongly influence the power relationship between provider and care seeker:

*“The RAMED changed a lot of things in our country. The patient’s perception of his dignity has changed. It has improved the care seeker’s confidence and consequently created a conflict with the caregiver. The RAMED policy also slightly changed the providers’ attitude: they have more respect for the patient now”* (R1).

Care seekers feel in a stronger position now that they are ‘entitled’, have the right to access (free) care. Respondents speak of more self-esteem and autonomy of the healthcare user, which the caregiver is not used to and might interpret as aggression or lack of gratitude. The result is a reciprocal and iterative process:

*“Patients become more aggressive. We too. We have not eaten, we are submerged by work and easily overlook some things. We make mistakes”* (R4).

An important nuance was made by respondents with regard to the healthcare users’ lower esteem of and trust in health providers. Respondents do not associate this phenomenon to FCH policies but mention the following causes:

*Generational changes:* Respondents observe a generational transformation at both patient and provider level:

*“Young people have another mentality. They assert their rights. Even the staff is not the same as in the past. They demand too much. Before, we worked without being aware of our rights”* (R18).

*Provider attitude and practices:* Respondents attribute the lack of esteem for health professionals to frequent corruption among doctors, absenteeism and poor contact with patients.

“*There is no longer the image of the doctor as the wise person, no longer the sublime glance the doctor had in the past” (R14).*

Esteem for the other does not change unilaterally:

*“People are less grateful and we are more mistrustful, for we do not work in a climate of trust and security. Hence, we do not give and do not draw the best out of ourselves”* (R4).

*Politics/media:* The political rhetoric and the media, loudly diffusing that everything is available in the public hospitals, play an important role.

*“People come with demands, and are disappointed upon arrival”* (R16).

*Urbanization:* In rural areas, patients need to create a link with only one, maximum two midwives.

*“They consider her a member of the family. Continuity of care is offered by only one midwife. At hospital level, there are several midwives. Patients cannot have trust in all”* (R12).

Finally, some respondents do not see any link between FHC policies and the patient-provider relationship:

“* My professional relationship has not changed. I always treat the patients correctly; it is how I was educated” (R17).*

They believe the mentality constraints can be overcome:

*“If we treat patients equally, we never have problems with them”* (R10).

Recommendations Formulated by Health Professionals

No respondent rejected the idea of vulnerable persons being exempted from payment for healthcare. Nevertheless, they argued that policymakers ideally first reflect on alternative efficient and rational processes, and ensure the means to finance FHC policies before actually introducing them. Although most respondents do not believe FHC to be sustainable, they are convinced that scaling back is impossible:

*“FHC policies are acquired rights now, socially irreversible”* (R14).

Yet, the following corrections and recommendations are proposed:

*Revision of FHC policy design:* In order to mitigate the impact of FHC on hospitals in densely populated areas, eg, on the availability and quality of care, most respondents suggest FHC to be provided only to the poor.

Respondents believe a basic health coverage regime such as the RAMED may replace the FOCP, on the condition that its eligibility criteria are revised in order to be sure to reach the truly vulnerable. Respondents also suggest revising the FOCP by: (1) including transport from home to the first level of care in the exempted package of care, since respondents believe this could shorten the first and second delay in seeking healthcare and therefore be an effective measure in combatting maternal morbidity and mortality; and (2) excluding elective caesarean sections from fee exemption, for “if it is scheduled, it is not urgent” (R18).

*Appropriate input to all elements of the health system:* Respondents regret the fact that effects of FHC policies on the health system have been insufficiently anticipated beforehand. Several recommendations involve an increased input into public health facilities: more and qualified human resources, a more complete and rapid cost recovery, a better availability of drugs, equipment and infrastructure (including beds and ambulances).

*“The citizen does not expect spectacular actions like organ transplants, but sustainable and outreach services. If we succeed in financing our public hospitals and provide them with sufficient human resources, we have nothing to envy the private sector for”* (R6).

Respondents request decision-makers to provide them with the means needed to offer the promised FHC.

*Quality enhancing measures:* To mitigate the collateral damage of FHC policies on the quality of care, respondents recommend to strengthen all levels of care from the bottom (primary healthcare) onward, in order to decongest the tertiary level. Reinforcement could be done by allocating more staff and material to each level, but also by task shifting, eg, training midwives in the use of ultrasound:

*“It increases the midwives’ and the doctor’s motivation. It is not the RAMED, but a climate of confidence and security, that will improve the quality of care”* (R4).

Furthermore, respondents suggest strengthening the existing quality surveillance tool in Moroccan hospitals, the *Concours Qualité,*^23^ by building on the expertise of health professionals. They argue that the current tool gives much importance to the patients’ opinions, while only health professionals really comprehend what quality is.

*“Patients only see the cleanliness”* (R3).

*Incentives to mitigate the risk of demotivation:* Respondents suggest strategies to motivate health professionals, among which offering training to healthcare providers and including them in discussions on health reform – for they consider themselves representatives of the community.

“*The authorities, at hospital and at state level, need to acknowledge health professionals, especially those who give a lot. We need to feel their recognition” (R9).*

Financial incentives are welcomed:

*“If midwives would be paid a symbolic price for every delivery they attend to, eg, 20 dirhams (two euros), you will see they will really give. A performance based payment that should also include the quality - for the mistake we make often is putting quantity before quality”* (R7).

## Discussion

Lipsky’s street level bureaucracy theory has been proven a useful theory to explain the modification or deficient implementation of a policy.^5,17,19,24,25^ Literature review and FEMHealth data, however, suggest that not all health professionals use their discretionary power. Some studies describe situations of conflict between different motivational influences. In Ghana, researchers categorized providers’ arguments pro et contra abortion in determinants related to public health knowledge, professional ethics, human rights, religion and morality, and concluded that it is exactly the complex interaction between these arguments that caused tensions and dilemmas among the providers.^25^ This study suggests that trying to marry contradictory personal determinants could be a potential reason for health workers to not implement a policy or to modify its contents or modalities.

Another potential reason for using one’s discretionary power is the potentially negative impact of the concerned policy on the work environment, for example on the workload or the revenue of health structures. A study of the impact of a fee exemption policy on health staff in Zambia found that health workers’ motivation was influenced in two ways: at the one hand the policy caused additional workload (through more clients and a lack of additional staff who used to be paid through user fees), decreased revenue for health centers (leading to stock rupture) as well as for providers (losing their bonus covered by user fees); on the other hand, providers claimed to be intrinsically motivated by the fact they were now able to provide care to all, including the poor.^26^ Similar contradictory motivational influences have been observed in our current study, explaining a certain duality in health workers’ perception and attitude towards the policy and the clients.

Erasmus points out that parts of the *street level bureaucracy* theory are overrepresented in studies that apply the concept to the health sector: the effects of workload and of limited work tools and resources on the discretion of providers-bureaucrats modifying the concerned policy, are rather well explained, while other work conditions (though described in the original theory of Lipsky) are not.^27^ Not all street level bureaucracy is grounded in intrapersonal factors; also contradictory organizational objectives may strongly influence the health workers’ attitude.^27^ A lack of guidelines for policy implementation, for example, can be perceived as a difficulty for the provider to achieve the objectives.^24^ Another example is a situation wherein objectives are contradictory and resources inadequate: eg, the government reimbursing health structures only partially for the free care they offer, Ghanaian providers deserted gratuity in an attempt to protect their institution from difficulties in paying creditors for drugs, consumables and other needs.^24^

Several studies demonstrate the association between process-related determinants and policy implementation.^6-8^ Effective policy implementation, however, is about more than clear policy formulation, excellent communication and strong collaboration between all stakeholders. At the point of service delivery, health workers may play a key role in shaping the way the policies are implemented.^17,19,25^ They are stimulated to do so when FHC policies strongly influence their professional environment and motivation.^24,26,28,29^ While effects of workload and of limited work tools and resources on the discretion of providers are rather well explained in studies applying Lipsky’s theory to the health sector, other effects, such as contradictory organizational objectives^24^ and the modification by providers of their conceptions and clients’ conceptions,^27^ are not.

Here, Franco’s model of motivational influences may be useful.^30^ It provides relevant insights in why the mechanism of street level bureaucracy is incited in one individual and not (or less) in another. Health providers are motivated at three levels: the individual level, the organizational level and the community level, including the interactions with patients. One is never influenced by a sole determinant; a mix of influences in interaction with an individual’s personality, at a specific moment in time, will determine the health workers’ attitude. Health sector reform, such as the FOCP, may influence intrapersonal determinants directly, or may do so through its effects on the organizational structures and interaction between community and patients – as elucidated by our study respondents. Increased patient influx without adequate means to cope with it may affect the individual’s ability – real and perceived – to carry out her/his tasks, and therefore stimulate or demotivate professionals in adoption of organization goals. Communities, too, may influence on health staff’s motivation through expectations regarding service delivery, through patient/provider interactions and through patients’ feedback on the providers’ performance.^30^ Health sector reform such as the introduction of a FHC policy, is likely to affect organizational systems and culture by changing the role of the community and patients. Study respondents indeed feel that their relationship to the care seeker has changed: he/she is more demanding for attention and quality care since he/she feels (and is) entitled to something. It comes with expectations – and disappointment, sometimes aggression, when those are not met. Intrapersonal values of the health professional, such as altruism, prestige, professionalism, security and unmet expectations are challenged by the patient’s demands. This, together with changes at organizational level (hospital) in the aftermath of FHC policies, influences health providers’ perceptions and attitude, and may affect their motivation.^28^

At first sight, few street level bureaucrats are spotted in the Moroccan health system. Are there none? There are. Yet, from the Moroccan example becomes clear that the footnotes in Lipsky’s theory should be read and respected: health workers are not the *de facto* decision makers that some make of them; public servants do not operate in a vacuum.^25^ Health workers are framed by context and culture, as well as by organizational and social hierarchies. This may explain why examples of the use of discretionary power are less manifest in politically authoritarian contexts with highly centralized health systems, such as the Moroccan one.

A first example of discretionary power put to use by Moroccan health professionals concerns the gratuity of care. Despite FHC policies being implemented “immediately and according to the letter,” informal conversations with care seekers and grey literature suggest that many Moroccans still pay for delivery-related acts and drugs. Once at ease in the conversation, study respondents confirm: patients pay for drugs that are not available, for gasoline for the ambulance, for ultrasound and so forth. Respondents state that corruption has become structural in Morocco because of a continuous imbalance between a limited offer and an unlimited demand - an imbalance reinforced by the FHC policies. At structural level, we see a lack of resources and an organizational culture based on negotiation and relational rather than procedural or formal arrangements. Personal factors play a role as well: to be able to give someone the appropriate care by purchasing the necessary treatment using the fee paid by the care seeker, is mentioned as motivating for the health professional confronted with stock shortages. Community and users share responsibility for this practice: according to respondents, patients ask to pay something in exchange for care of *good* quality. Finally, as suggested by one of the study respondents, the cultural and political context, not spared from corruption, may have an exemplary role and as such enhance, or legitimize, this practice at hospital level. In order to avoid health workers abusing, FHC policies should be precisely prepared with elucidation of revenue-raising modalities and purchasing arrangements that guarantee best value for money – identified through health technology assessments. Using a system approach is an absolute condition for an FHC policy to be an effective step towards universal health coverage.

A second example of discretionary power is provided by the way maternal death audits are done. Being an important mortality surveillance (hence quality improvement) tool, clinical audits of maternal deaths are systematically put in place since 2008.^2^ Interviewees declare those audits not to be truthful. Health workers exaggerate the health status of the woman when in their facility, so it seems her health only deteriorated in the referral hospital. Fear and an inadequate accountability model may be at the basis of this practice. Health professionals express to be scared of being declared responsible for the patient’s death, and of the consequences of such judgment. Belgian jurisdiction may provide an exemplary alternative: it increasingly holds the system (hospital) and not the health professional responsible for medical erring. The patient rights’ law of 2002 foresees in a central hospital liability, whereby the applicant does not need to identify the health workers responsible.^31^ The hospital can be summoned if the damage occurred in the hospital where the health workers were practicing. After all, often it is not clear whether and to what extent a health professional has erred. Moreover, if a fault or negligence is proven, chances are that it is actually a series of errors, hence involving several professionals who all represent only one cogwheel in a complex system. Maternal death audits can only be effective when implemented in a climate of confidence. Blaming and judgment weaken this instrument’s effectiveness. In order to avoid health workers to abuse this tool and as such hollow its efficacy, we strongly recommend creating a confidential and secure environment, in which health providers find the space to work in alignment with their professional ethics, to react timely, to collaborate and to take responsibility.

The examples above demonstrate that reasons for not implementing a policy as planned are variable and not necessarily linked to intrapersonal factors. Providers also pursue organizational goals and seek to satisfy the community of which they are member. A key notion in our study seems to be the frontline responsibility. Who bears the responsibility of the state failure to comply with its promised free care, eg, when a health provider is unable to provide a patient with the necessary treatment due to stock rupture? What would be the conditions for ‘frontliners’ (health professionals) to bear this responsibility? The absolute minimum would be: not feeling endangered. Better would be: feeling protected. In Maslow’s hierarchy of needs, indicating how people in general achieve a sense of satisfaction, “safety and security” comes second, after the need for food and water. Higher needs (social, esteem and status, self-actualization) cannot be satisfied if lower-level needs are not met.^32^ Not feeling protected then leads to the impossibility to feel satisfied, even if esteem and recognition would be experienced. Another interesting motivational theory is Adam’s equity theory, suggesting that employees are more or less motivated based on the degree to which they believe they are being treated fairly, particularly by their supervisors and managers.^33^ When employees feel they are putting in more effort than their peers, yet do not believe to be appropriately rewarded for that effort, they are likely to be unmotivated. This accounts as well for employees who feel their level of pay is not equitable compared to other employees or other companies. Both examples were found among the respondents of this study.

Can FHC policies be a relevant add-on to basic health coverage schemes? Study respondents believe alternatives could be explored before turning to FHC policies. If put in place, FHC policies need to be carefully prepared for two reasons: in order to achieve their objective – making access of the poor to (quality) care possible – and in order to mitigate unwanted side effects, such as shortage of human, material and financial resources, deteriorating work conditions and decreased motivation in health professionals. Among accompanying measures to optimize policy implementation is the reinforcement of workforce motivation. Since health professionals are both intrinsically and extrinsically motivated, financial incentives are not sufficient as a reinforcing measure.^34^

### Strengths and Limitations of the Study

This study has a number of limitations. In-depth interviews were held in the native language of neither interviewer nor respondents. We mitigated through the use of an interpreter in two interviews, and through discussion of study results with fellow researchers, most of whom have extensive knowledge of the Moroccan context and culture. Secondly, we built the study on a relatively small number of interviews. Moreover, few gynaecologists were available. An added value was provided by the self-expressed interest of high-level managers in study participation. Most of hospital managers being specialized doctors, it partly compensates the lack of this profile among the health providers. Lastly, healthcare users were not interviewed. Additional qualitative research focusing on users’ perceptions is needed to fully understand the complex interactions within the health system, and how they are influenced by FHC policies.

## Conclusion

Health professionals appreciate FHC policies from an ethical point of view. However, they question the effectiveness of such policies if not accompanied by the necessary investments, both in health and associated sectors. They argue that fee exemption may negatively influence service delivery and public health worker motivation, potentially leading to perverse effects. Health professionals mention an increased workload, insufficient resources, infrastructural constraints and changes in the patient/provider relationship as main causes for these negative influences. Study respondents confirm that health reform, such as FHC policies, generates a power shift in the patient/provider relationship. Demotivating for some, this may also create new opportunities, such as more patient autonomy and increased participation.

## Acknowledgements

This work was supported by the FP7-PEOPLE-2013-IRSES Marie Curie Actions project funded by the European Union (Grant Agreement No. 612216). The funder had no role in the study design, data collection and analysis, decision to publish, or writing of the manuscript. The corresponding author is very grateful to Prof. Dr. Vincent De Brouwere for having made this study possible in organisational terms and for his critical review of the article. In addition, the authors are grateful to Dr. Amina Essolbi and Dr. Bouchra Assarag, for having facilitated the administrative and practical aspects of the study implementation in the selected districts.

## Ethical issues

Our study has been approved and registered (B670201628352) by the ethical committee of the University of Ghent, Ghent, Belgium. It has received additional approval by the Institutional Review Board of the Institute of Tropical Medicine, Antwerp, Belgium and the ethical committee for biomedical research of the University of Rabat, Rabat, Morocco.

## Competing interests

Authors declare that they have no competing interests.

## Authors’ contributions

KV designed the study with critical input by PD. Data were collected by KV and analysed by KV, FD, and PD. The manuscript was drafted by KV with contributions of FD and PD. BM critically revised the manuscript. Supervision of the study was done by PD and BM.

## Authors’ affiliations

^1^Department of Public Health, Institute of Tropical Medicine, Antwerp, Belgium. ^2^Department of Public Health and Primary Care, Ghent University, Ghent, Belgium.

## Key messages

**Implications for policy makers**

- A more inclusive and bottom-up policy design may improve policy adoption by health professionals.
- Policy-makers seizing the opportunity, embedded in free healthcare (FHC) policies, to enhance patient’s participation in the healthcare system and patient autonomy, may contribute constructively to a better performing health system.

**Implications for public**

Health professionals appreciate free healthcare (FHC) policies from an ethical point of view. However, such policies may, if not accompanied by the necessary investments in health and associated sectors, become a significant burden for the health system and negatively affect its performance. Health professionals express significant influences of FHC policies on their working environment and conditions (higher workload and scarcity of resources) and on the patient/provider relationship, both of which may have detrimental effects on health workers’ motivation. A mix of motivational determinants incites health workers on their turn to influence policy implementation. Including professionals in policy design and planning, may optimize health reform processes and results.
